# Comparative efficacy of microwave ablation and radiofrequency ablation for treating metastatic liver cancer: a systematic review and meta-analysis

**DOI:** 10.3389/fonc.2024.1473780

**Published:** 2024-10-30

**Authors:** Zheng Li, Tingting Yan, Xiujun Cai

**Affiliations:** ^1^ Department of General Surgery, Sir Run Run Shaw Hospital, Zhejiang University School of Medicine, Hangzhou, China; ^2^ National Engineering Research Center of Innovation and Application of Minimally Invasive Instruments, Hangzhou, China; ^3^ Zhejiang Minimal Invasive Diagnosis and Treatment Technology Research Center of Severe Hepatobiliary Disease, Hangzhou, China

**Keywords:** meta-analysis, systematic review, metastatic liver cancer, microwave ablation, radiofrequency ablation

## Abstract

**Objective:**

This study aims at evaluating and juxtaposing the efficacy of radiofrequency ablation (RFA) and microwave ablation (MWA) for hepatic metastases treatment.

**Methods:**

We undertook an extensive literature search across the Cochrane Library, Web of Science, Embase, PubMed, CNKI, and databases for studies published up to December 2023, assessing the outcomes of RFA versus MWA in hepatic metastases treatment. Studies were included or excluded based on established criteria. Continuous variables were analyzed with the aid of the weighted mean difference (WMD) and its 95% confidence interval (CI), while the odds ratio (OR) with its 95% CI was utilized for dichotomous variables. Data were processed by use of STATA 17.0 software. Key outcomes assessed included ablation time, post-operative local tumor progression (LTP), disease-free survival (DFS), and post-operative complications (POCs).

**Results:**

Seven studies, comprising 357 patients undergoing MWA and 452 patients undergoing RFA, fulfilled the inclusion criteria. As unveiled by the meta-analysis, RFA and MWA did not significantly differ in ablation time, DFS, and POCs. Nonetheless, MWA resulted in a strikingly reduced rate of post-operative LTP versus RFA.

**Conclusion:**

MWA offers superior control over post-operative LTP, suggesting better overall efficacy in hepatic metastases treatment compared with RFA.

**Systematic Review Registration:**

https://www.crd.york.ac.uk/prospero/, identifier CRD42023385201.

## Introduction

Liver cancer (LC), a prevalent malignancy worldwide, is distinguished as primary and metastatic types. The cells from which primary LC originates are hepatocytes or those of the intrahepatic bile ducts, whereas metastatic LC, also known as secondary LC, occurs when malignant tumors from other body sites invade the liver. Gastrointestinal cancers are the most typical origin of hepatic metastases, taking up 50%–60% of cases (1). Advanced-stage diagnosis of liver metastases is not rare, leading to poor prognosis, with the five-year survival rate for gastric cancer (GC) with liver metastases falling below 10% (2). Surgery continues to be the mainstay treatment for liver metastases. However, many patients are ineligible for surgery due to the number, location, and complications of the metastases. For example, only 20% of GC cases and 10%–20% of CC cases of liver metastases undergo surgery (3). Cases of liver metastases who skip surgery have a five-year survival rate of under 5%.

For inoperable tumors, thermal ablation combined with systemic chemotherapy can significantly improve prognosis (4). Thermal ablation also offers the advantage of being repeatable to address local progression (5). It is extensively utilized for primary hepatocellular carcinoma (HCC). Both radiofrequency ablation (RFA) and microwave ablation (MWA) operate on the principle of local thermal injury. Compared with RFA, RFA works by conducting radiofrequency current to the tumour site, using electrode needles to deliver high-frequency current into the tissue, and using the thermal effect of the current to achieve tumour ablation.MWA, on the other hand, transmits electromagnetic waves through a microwave antenna, and the microwave field can cause water molecules to rotate at high speed, generating frictional heat (6). MWA theoretically provides larger ablation volumes, higher tissue temperatures (7), more uniform cell death, shorter treatment times, a lower risk of heat sink effect, and enhanced local tumor control rates. Research has shown that MWA requires more energy to reach an equivalent ablation volume in liver metastases compared with HCC. Previous meta-analyses have primarily focused on the comparison of ablation techniques for HCC. However, due to the biological differences between primary and metastatic LCs, the ideal thermal ablation method for liver metastases remains controversial (8). This research systematically reviews the literature to compare the effectiveness of MWA and RFA in treating liver metastases in the short term.

## Materials and methods

### Approach to literature search

A detailed literature review and meta-analysis was implemented *as per* the PRISMA guidelines and the Cochrane Handbook for Systematic Reviews of Interventions. Independent searches by the authors in the Cochrane Library, CNKI, Web of Science, Embase, and PubMed databases were implemented for literature published up to December 2023 that compared the efficacy of radiofrequency ablation (RFA) and microwave ablation (MWA) in liver metastases therapies. Search terms included “microwave ablation,” “liver metastases,” and “radiofrequency ablation.” This search was unrestricted by language but limited to human studies. The PubMed search strategy included keywords such as “microwave ablation,” “radiofrequency ablation,” and “liver neoplasms.” Additionally, all retrieved articles’ reference lists were checked to uncover additional pertinent research.

### Inclusion and exclusion criteria

The following were the criteria for inclusion: 1) studies featuring subjects diagnosed with liver metastases, 2) those evaluating the efficacy of MWA versus RFA, and 3) those reporting at least one relevant outcome metric, such as operative time, ablation efficacy, post-operative outcomes, or complications.

Exclusion criteria were shown below: 1) studies not directly comparing MWA with RFA, 2) those lacking extractable data, or 3) non-original research articles, including conference abstracts, review articles, case reports, and letters.

### Study selection and data extraction

Subsequent to duplicate removal, Zheng Li and Tingting Yan executed an independent screening of the abstracts and titles *as per* the inclusion criteria respectively. For articles with unclear eligibility, the full text was reviewed independently by two researchers, and any disagreements were resolved through discussion. Data collection employed a predefined extraction form, capturing information such as the first author, number of subjects, age, publication year, sex, country, tumor size, follow-up span, and relevant outcome metrics. Primary outcomes included ablation time and post-operative outcomes (disease-free survival [DFS], total post-operative complications, and local tumor progression [LTP]).

### Assessment of methodological quality

Assessment of the included studies’ methodological quality was accomplished by harnessing the modified Newcastle-Ottawa Scale (NOS), evaluating three domains: patient enrollment, group comparability, and results evaluation. A scoring system from zero to nine was utilized for each study, where scores of 7 and above signified high quality.

### Data processing

Implemented with the aid of Review Manager 5.4 software, the meta-analysis examined continuous variables with weighted mean differences (WMD) and 95% confidence intervals (CI) and dichotomous variables with odds ratio (OR) and 95% CI. Statistical significance was determined by P < 0.05.

To get the measure of statistical heterogeneity among studies, the chi-square test was utilized, with a significance cutoff of P < 0.05. The I² statistic quantified heterogeneity, with I² > 50% indicating substantial heterogeneity. If I² is high (e.g. >50 per cent), it should be assessed using a random-effects model. Conversely, use a fixed-effects model (9, 10).Sensitivity analysis, involving the sequential exclusion of each study, was executed to assess the results’ robustness and identify potential sources of heterogeneity. When the samples in Meta-analysis are less than 10, the trim and fill correction is more likely to produce inaccurate results due to random variation (11, 12). Therefore, we used Egger’s test and Begg’s test to assess the risk of publication bias. Generated by Review Manager 5.4, funnel plots were utilized alongside Egger’s and Begg’s tests in Stata 17.0 software for publication bias assessment.

## Results

### Study selection and characteristics

**Figure 1:**
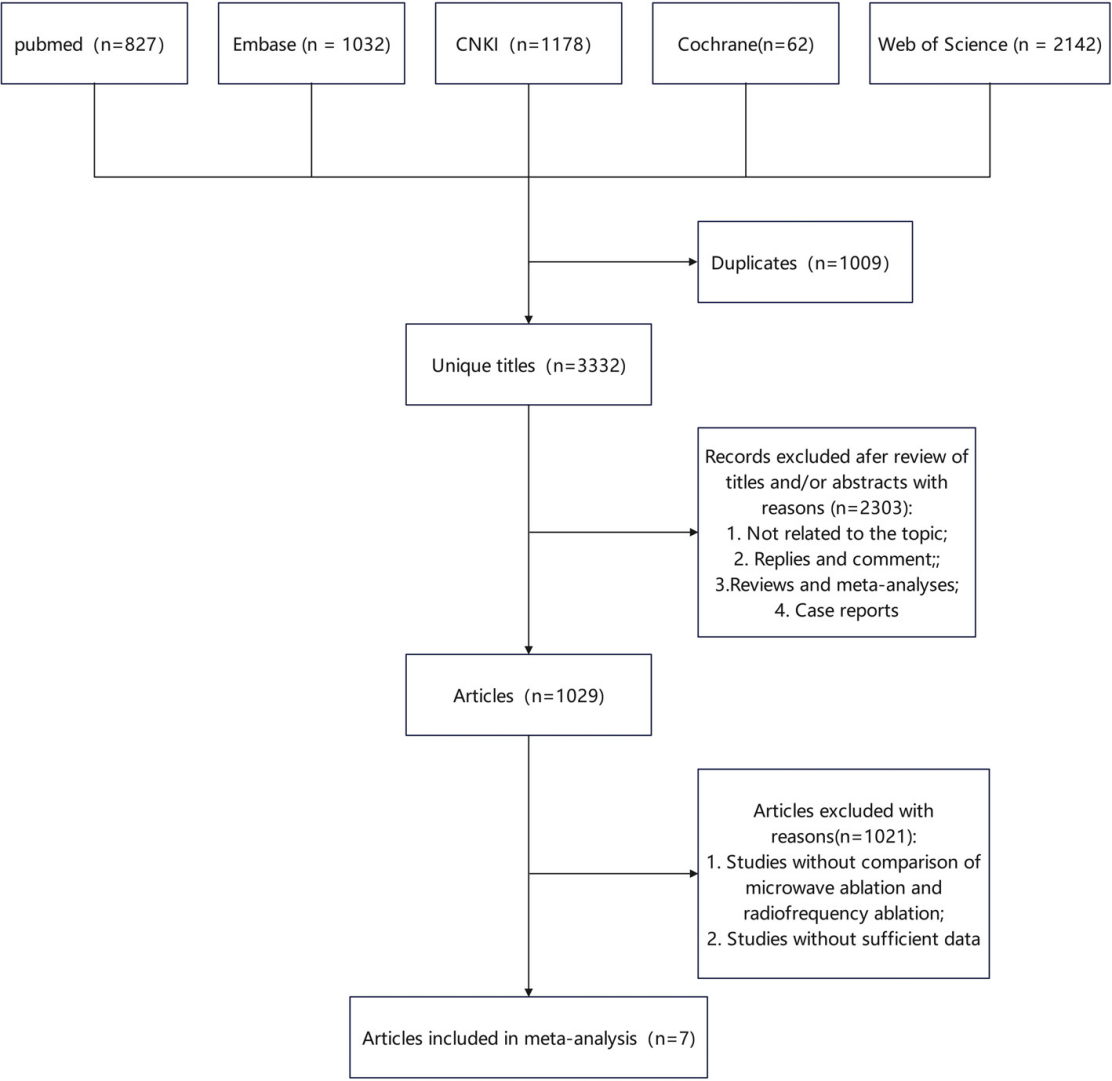
Flowchart of the systematic search and selection process.

**Figure 1** depicts the selection process of studies. A systematic literature search identified 4,341 potentially relevant studies from various databases: PubMed (n = 827), Embase (n = 1,032), Web of Science (n = 2,142), Cochrane Library (n = 62), and CNKI (n = 1,178). After removing duplicates, 3,332 studies remained. Based on the type of work, 2,303 articles were excluded, including reviews, conference papers, and other non-eligible literature. Ultimately, the analysis included seven studies that met the inclusion criteria (13–19). All seven studies were retrospective cohort studies, with six studies scoring 9 on the NOS and one study scoring 8, indicating high methodological quality. **Table 1** presents a summary of the included studies’ characteristics. The MWA group included 357 patients, while the RFA group included 452 patients. These studies were conducted in the USA (three studies), Netherlands (two studies), Germany (one study), and China (one study).

**Table 1:** Baseline characteristics of include studies.

Author	Region	Year	Study period	Ablation technique	Total patients	Mean age years	Gender male/famle	Tumor mumber
Correa-Gallego	USA	2014	2008-2011	MWA	67	55	NR	127
				RFA	67	56	NR	127
Tilborg	Netherlands	2016	2001-2014	MWA	15	63	12/3	32
				RFA	96	61	60/36	139
Yang	China	2017	2010-2016	MWA	71	51	49/22	121
				RFA	108	50	68/40	188
Takahashi	USA	2018	2014-2018	MWA	51	NR	33/18	121
				RFA	54	NR	33/21	155
Shady	USA	2018	2009-2015	MWA	48	NR	35/13	60
				RFA	62	NR	38/24	85
Vogl	Germany	2022	2014-2016	MWA	26	62.7	14/12	NR
				RFA	24	63.3	13/11	NR
Krul	Netherlands	2022	2013-2018	MWA	79	61.1	42/37	193
				RFA	41	63	22/19	98

Screening these seven studies’ reference lists manually did not reveal any additional suitable studies.

### Meta-analysis results

#### Ablation time

**Figure 2:**
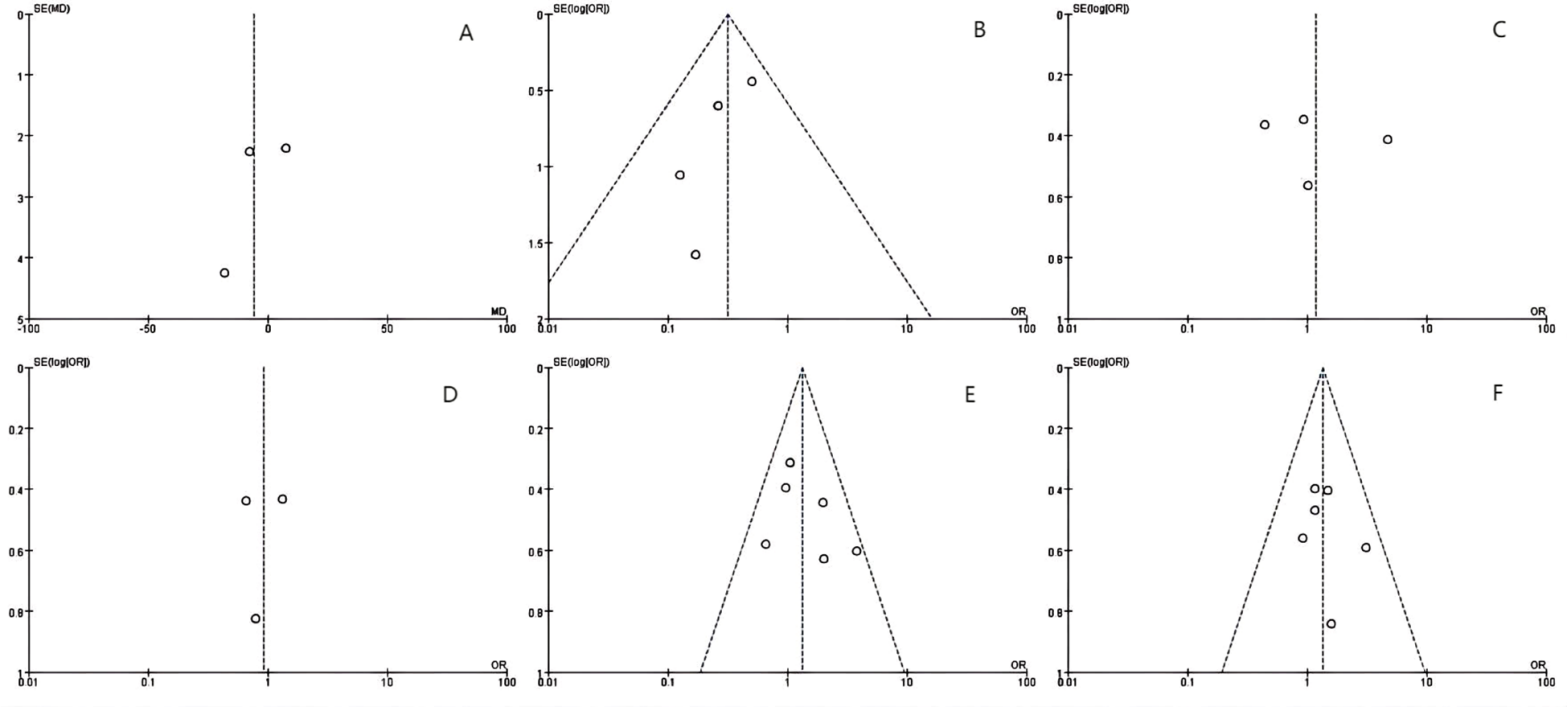
The Begg’s tests indicates the absence of publication bias: **(A)** ablation time, **(B)** post-operative local tumor progression (LTP) by number of patients, **(C)** LTP by number of tumors, **(D)** disease-free survival (DFS)for 1 year, **(E)** DFS for 2 years and **(F)** post-operative complications.

**Figure 3:**
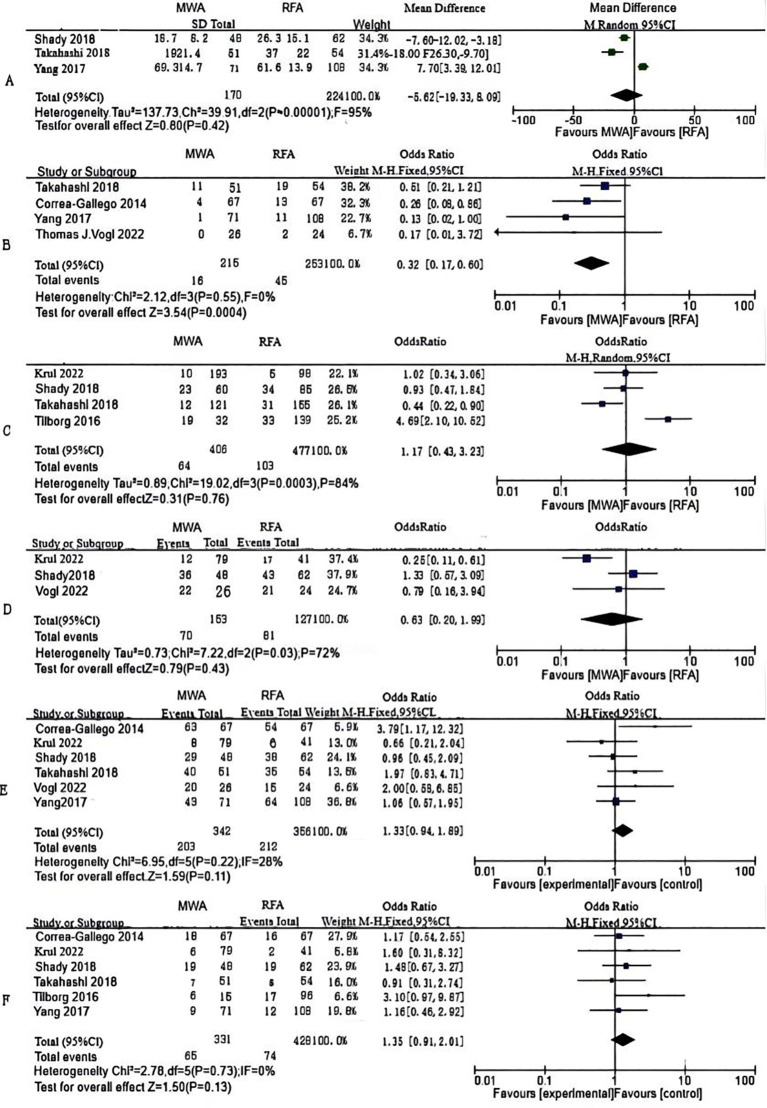
Forest plots of comparative data: **(A)** ablation time, **(B)** post-operative local tumor progression (LTP) by number of patients, **(C)** LTP by number of tumors **(D)** disease-free survival (DFS)for 1 year, **(E)** DFS for 2 years and **(F)** post-operative complications.

Ablation time data were extracted from three studies, comprising 394 patients (MWA group: 170; RFA group: 224). *As per* the combined analysis, the intergroup difference in ablation time was not significant (WMD: -5.62; 95% CI: -19.33, 8.09; P = 0.42), exhibiting statistically noticeable heterogeneity (I² = 95%, P < 0.00001). The funnel plot suggested slight publication bias, but Egger’s (P = 0.534) and Begg’s tests (P = 0.296, **Figure 2A**) did not reveal statistically striking publication bias (**Figure 3A**).

#### Post-operative outcomes

LTP, the reappearance of tumor lesions at the ablation zone margin, was analyzed based on data from four studies. Analysis showed a striking lower incidence of tumor progression in the MWA group (OR: 0.32; 95% CI: 0.17, 0.60; P = 0.0004), with no significant heterogeneity (I² = 0, P = 0.55). Although the funnel plot indicated publication bias, Egger’s (P = 0.167) and Begg’s tests (P = 0.734, **Figure 2B**) unveiled statistically remarkable publication bias (**Figure 3B**).

Data on the number of tumor nodules exhibiting progression were available from four studies. The combined analysis showed no significant inter-group discrepancies (OR: 1.17; 95% CI: 0.43, 3.23; P = 0.76), alongside statistically pronounced heterogeneity (I² = 84%, P = 0.0003). The funnel plot suggested slight publication bias, but Egger’s (P = 0.747) and Begg’s tests (P = 0.734, **Figure 2C**) uncovered no statistically significant publication bias (**Figure 3C**).

#### Survival outcomes

We made measurements of one- and two-year DFS rates. Three studies reported one-year DFS, which uncovered no statistically pronounced discrepancy between the MWA and RFA groups (OR: 0.93; 95% CI: 0.53, 1.63; P = 0.79), alongside no striking heterogeneity (I² = 0, P = 0.52). The funnel plot unveiled no significant publication bias, corroborated by Egger’s (P = 0.869) and Begg’s tests (P = 1.000, **Figure 2D**) (**Figure 3D**).

Six studies reported two-year DFS. The combined analysis showed that the MWA and RFA groups had no striking discrepancy in two-year DFS between (OR: 1.33; 95% CI: 0.94, 1.89; P = 0.11), alongside no noticeable heterogeneity (I² = 28%, P = 0.22). The funnel plot suggested slight publication bias, but Egger’s (P = 0.353) and Begg’s tests (P = 0.707, **Figure 2E**) uncovered statistically significant publication bias (**Figure 3E**).

#### Complications

Six studies reported post-operative complications. The combined analysis uncovered no striking inter-group discrepancy in the complication rates (OR: 1.35; 95% CI: 0.91, 2.01; P = 0.13), with no significant heterogeneity (I² = 0, P = 0.73). The funnel plot as well as Egger’s (P = 0.553) and Begg’s tests (P = 0.707, **Figure 2F**) revealed statistically pronounced publication bias (**Figure 3F**).

#### Sensitivity assessment

**Figure 4:**
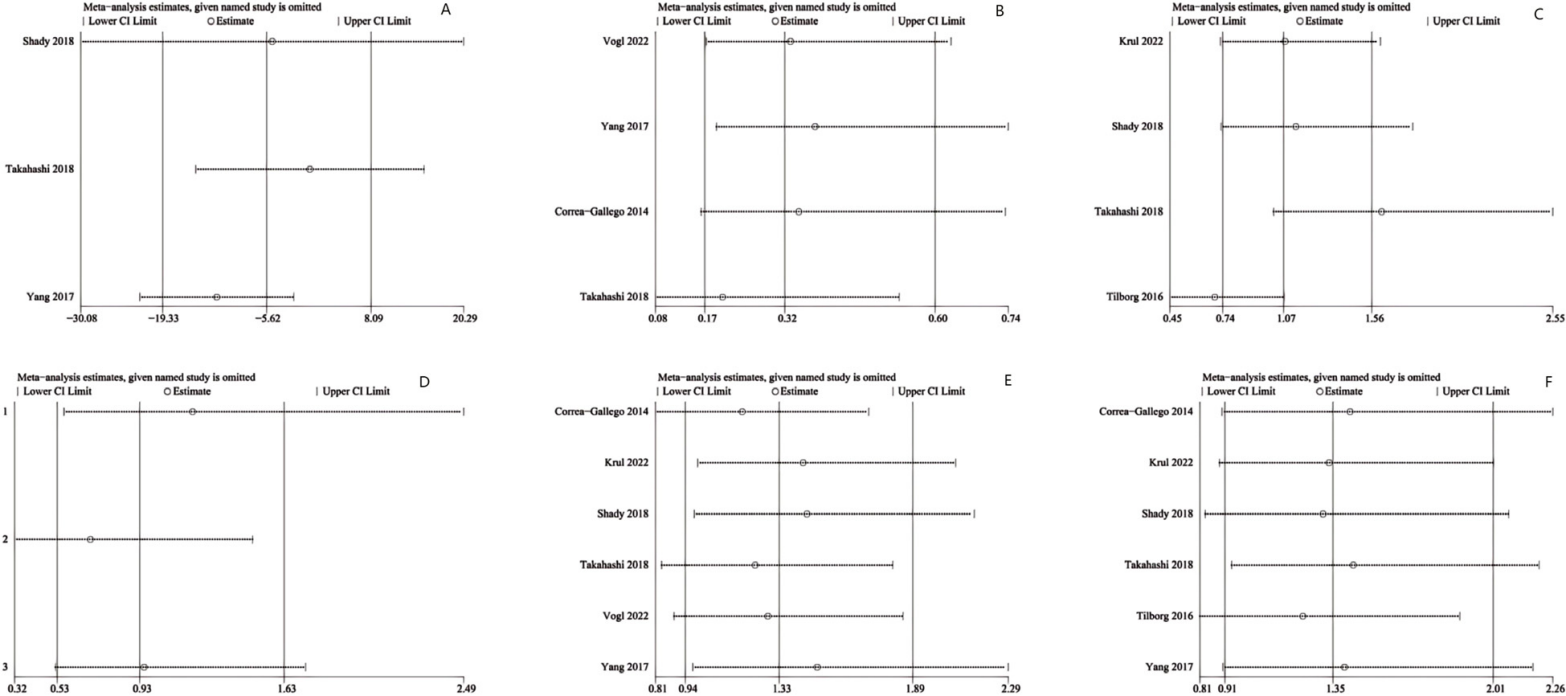
Sensitivity analysis of **(A)** ablation time, **(B)** post-operative local tumor progression (LTP) by number of patients, **(C)** LTP by number of tumors, **(D)** disease-free survival (DFS) for 1 year, **(E)** DFS for 2 years and **(F)** post-operative complications.

Sensitivity analyses were performed for ablation time (**Figure 4A**), post-operative LTP (**Figures 4B, C**), DFS (**Figures 4D, E**), and post-operative complications (**Figure 4F**) *via* sequential exclusion of each study to gauge the impact on the pooled WMD or OR and CI. The sensitivity analysis showed stable results for LTP, DFS, and post-operative complications, with the pooled estimates remaining unchanged after excluding any single study. However, for ablation time, study exclusion by Yang et al. (15) changed the result from non-significant to significant (WMD: -12.18; 95% CI: -22.30, -2.06; P = 0.02), with a marked reduction in heterogeneity (I² = 79%, P = 0.03), indicating instability in this metric.

## Discussion

This study focuses on the comparative effectiveness of MWA and RFA in treating liver metastases. We included seven studies to evaluate differences in operative time, LTP rate, DFS, and complication rates between the two techniques.

Given the relatively recent adoption of MWA, there are fewer studies directly comparing its efficacy to RFA. Our meta-analysis evaluated ablation time, LTC, DFS, and post-operative complications. For ablation time, the combined data from three studies unveiled that MWA did not pronouncedly differ from RFA. However, sensitivity analysis indicated potential publication bias. Two studies reported shorter operative times for MWA (16, 17) while another study provided only median ablation times (with medians of 70 for MWA and 60 for RFA) (15), showing a narrower time range for MWA. This finding suggests that publication bias might arise from variations in data reporting. Additionally, some studies noted that MWA equipment requires minimal training for surgeons, potentially facilitating broader adoption compared with RFA (19). The operational ease of both techniques should be the emphasis of future research, as this can significantly impact their clinical implementation.

Regarding LTP, one-year follow-up results indicated that the MWA group displayed strikingly lower LTP rates versus the RFA group, suggesting a potentially broader ablative margin with MWA.This advantage may be attributed to MWA’s ability to penetrate tissues more effectively (20) near blood vessels and improve tissue conductivity (17), a limitation for RFA (21) due to its outward-to-inward heat conduction pattern (22, 23). MWA’s capability for multi-probe operations (24) and creating larger ablation zones (25) further contributes to its efficacy. However, the two-year follow-up highlighted no outstanding inter-group discrepancy (P = 0.76). This result may be caused by RFA’s limitations in ablating tumors proximal to large blood vessels and the rapid growth of primary tumors in cases of liver metastases. For highly malignant and poor prognosis tumors, the ability of MWA to reduce the one-year local progression rate demonstrates a superior ablation effect to RFA.However, it should also be noted that in clinical practice, tumour size and location have a significant impact on the clinician’s choice of radiofrequency ablation or microwave ablation. RFA is usually used for smaller tumours, especially those located away from large blood vessels, because of its more pronounced heat sink effect. MWA, on the other hand, is particularly suitable for treating larger tumours or tumours located near large blood vessels due to its higher power output and less heat sink effect. This difference in clinical decision-making may result in different for LTP and ultimately interfere with the results of Meta-analysis (26, 27).

Moreover, we compared one-year and two-year DFS rates. Yang’s study (15) noted that MWA creates larger ablation zones, leading to lower LTP rates and improved long-term outcomes. Only three studies reported one-year DFS, with two studies suggesting higher DFS rates following MWA implementation. However, *as per* the combined analysis, the MWA and RFA groups exhibited no noticeable discrepancy (P = 0.79). Six studies reported two-year DFS, which denoted no inter-group discrepancy (P = 0.11). The limited number of studies and the poor overall condition of cases undergoing liver metastases, coupled with the rapid growth of primary tumors, may obscure the efficacy differences between the two ablation techniques.

For post-operative complications, six studies were analyzed, revealing no noticeable discrepancy between the MWA and RFA groups (P = 0.13). Nonetheless, five of these studies indicated more complications in the RFA group, consistent with previous meta-analyses (28). Considering MWA’s advantages of minimal heat sink effect, regular ablation zones, and suitability for ablation near large blood vessels, increasing the sample size might reveal significant differences in complication rates.

In conclusion, although two-year LTP and DFS rates displayed no observable discrepancies between the two ablation methods, MWA demonstrates advantages such as shorter operative time, higher one-year local control rates, and fewer complications. These findings suggest that MWA may be a more ideal choice for treating liver metastases. Given the limitations of the small sample size of included studies, additional robust studies are pivotal to validate these findings. Additionally, factors such as ease of operation and cost-effectiveness of both ablation techniques are important considerations for their broader clinical application and warrant further investigation.

## Data Availability

The original contributions presented in the study are included in the article/supplementary material. Further inquiries can be directed to the corresponding authors.
